# Impact of Global Climate Change on the European Barley Market Requires Novel Multi-Method Approaches to Preserve Crop Quality and Authenticity

**DOI:** 10.3390/foods10071592

**Published:** 2021-07-08

**Authors:** Stefan G. Bindereif, Felix Rüll, Peter Kolb, Lucas Köberle, Holger Willms, Simon Steidele, Stephan Schwarzinger, Gerhard Gebauer

**Affiliations:** 1 BayCEER—Laboratory of Isotope Biogeochemistry, University of Bayreuth, Universitätsstraße 30, 95447 Bayreuth, Germany; stefan.bindereif@uni-bayreuth.de; 2 NBNC—North Bavarian NMR Centre, University of Bayreuth, Universitätsstraße 30, 95447 Bayreuth, Germany; Felix.Ruell@uni-bayreuth.de (F.R.); peter.kolb.mail@googlemail.com (P.K.); simon-steidele@outlook.de (S.S.); 3 ALNuMed GmbH, Gottlieb-Keim Straße 60, 95448 Bayreuth, Germany; lucasausberg@gmx.de; 4 IREKS GmbH, Lichtenfelser Straße 20, 95326 Kulmbach, Germany; holger.willms@ireks.com

**Keywords:** barley, stable isotopes, proton nuclear magnetic resonance, Fourier transform near-infrared spectroscopy, drought, climate change, Europe, Germany, multi-method approach, beer

## Abstract

Most recently in 2018 and 2019, large parts of Europe were affected by periods of massive drought. Resulting losses in cereal yield pose a major risk to the global supply of barley, as more than 60% of global production is based in Europe. Despite the arising price fluctuations on the cereal market, authenticity of the crop must be ensured, which includes correct declaration of harvest years. Here, we show a novel approach that allows such differentiation for spring barley samples, which takes advantage of the chemical changes caused by the extreme drought. Samples from 2018 were successfully differentiated from those of 2017 by analysis of changes in near-infrared spectra, enrichment in the isotope ^13^C, and strong accumulation of the plant-physiological marker betaine. We demonstrate that through consideration of multiple modern analysis techniques, not only can fraudulent labelling be prevented, but indispensable knowledge on the drought tolerance of crops can be obtained.

## 1. Introduction

Recent climate changes in Europe are characterized by frequent and severe weather extremes, which pose a major threat to agricultural production and are predicted to increase in the near future [1,2]. Particularly during the last two decades, most parts of Europe were impacted by record-breaking droughts leading to losses in both crop yield and quality. Global warming poses a serious challenge to the global barley (*Hordeum vulgare* L.) supply, as more than 60% of the world’s barley production is provided by Europe, most of it grown under rainfed conditions [3]. This crop is mainly used as malting barley for the production of alcoholic beverages and for feeding animals. European beverage producers typically use spring barley for malting due to its low protein content and thus more fermentable portion of sugar. However, spring barley has its most sensitive periods during flowering and grain filling around the summer months, hence there is a high risk of yield loss and crop failure in extremely dry years [4]. In 2018, Germany, the largest producer of spring barley in Europe, experienced its warmest year ever recorded in the 138 years of modern temperature records, ultimately resulting in the lowest cereal yield since 1994 and creating existence-threatening conditions for many farmers across the country [5]. Subsequently, farmers were forced to mix low-grade spring barley batches and marketable grains from previous harvest years to meet the quality requirements of the customers. However, malthouses often refuse to accept deliveries of blends from two harvest years, consequently the product can only be offered as significantly less profitable feed barley [6]. Such stages of emergency increase the risk of economically motivated food fraud. The likelihood of these scenarios gains in importance when considering a recently published model by Xie et al., who found that the effects of concurrent drought extremes will cause a large decrease in global barley supply under forecasted climate conditions [7]. To preserve transparency in such difficult periods, the development of a method that is capable of classifying spring barley by harvest year is indispensable. Here, we assess the potential of distinguishing spring barley grain samples according to the very different harvest years 2017 and 2018. We looked at two varieties, namely the modern and high-yielding cultivar ‘Quench’ (*H. vulgare* L. cv. Quench, year of approval: 2006) and the comparatively old cultivar ‘Steffi’ (*H. vulgare* L. cv. Steffi, year of approval: 1989). As results produced by a single analytical method often do not provide enough information for an adequate evaluation, we have developed a novel methodology using fused data from stable isotope analysis (IRMS) and chemical fingerprints provided by Fourier-transform infrared spectroscopy (FT-NIR). Furthermore, metabolite profiles produced by proton nuclear magnetic resonance (^1^H-NMR) were applied to substantiate the findings through identification of stress-related plant physiological parameters. For the present work, we hypothesize that the severe drought events of 2018 created systematic variability in the chemical makeup of barley grains that, when evaluated by our multi-methodological approach, should allow effective separation from samples of 2017. Based on previous works found in literature, we further hypothesize that the older barley cultivar ‘Steffi’, though less yielding, should be more drought-tolerant than the modern cultivar ‘Quench’.

## 2. Materials and Methods

### 2.1. Origin and Storage of the Spring Barley Samples

Eighty-six spring barley samples of two different varieties (*H. vulgare* L. cv. Quench; *H. vulgare* L. cv. Steffi) and from two consecutive harvest years (Quench, n_2017_ = 19, n_2018_ = 20 and Steffi, n_2017_ = 22, n_2018_ = 25) have been provided by IREKS GmbH (Kulmbach, Germany). As the focus of this study was put on describing the effects of years with extreme drought, we were provided with barley grains grown under real conventional farming conditions rather than controlled field experiments. All plants were cultivated in the same region by an agricultural cooperative of the district Soemmerda (Thuringia, Germany) on loamy soil at 140 m elevation to eliminate geographic effects. Samples were received as whole grains and stored in vacuum-sealed bags at room temperature until further processing.

### 2.2. Weather at the Sampling Site

Weather data were retrieved from the Climate Data Center of the German Meteorological Service [8]. The De Martonne aridity index (I_DM_) was used as an indicator of drought, which is calculated according to the following equation:

(1) $$I_{\mathrm{DM}}=\mathrm{RR}/\left(\mathrm{TM}+10\right)$$

where RR is the mean annual precipitation in mm and TM is the mean air temperature in °C [9]. I_DM_ values below 30 correspond to a humid to Mediterranean habitat, while regions with I_DM_ values below 20 are characterized by semi-dry to dry conditions.

### 2.3. Isotope-Ratio Mass Spectrometry (IRMS)

For measurement of natural isotope abundances of the elements C, N, and O, around 4 g of grains were ground to wholemeal flour in a ball mill (MM400, Retsch GmbH, Haan, Germany) at 30 Hz for 2 min. The sample material was dried in a drying oven at 105 °C to constant weight and stored in a desiccator until analysis. For carbon and nitrogen measurements, 2.8–3.5 mg of the powder was weighed into tin capsules, while for oxygen measurements 0.5–1 mg was weighed into silver capsules using a microbalance (CPA2P, Sartorius, Göttingen, Germany).

Carbon and nitrogen stable isotope ratios were measured using an elemental analyzer (NA 1108, Carlo Erba Instruments, Milan, Italy) coupled to a continuous flow isotope-ratio mass spectrometer (delta S, Finnigan MAT, Bremen, Germany) via a ConFlo III open-split interface (Thermo Fisher Scientific, Bremen, Germany) as described by Bidartondo, Burghardt, Gebauer, Bruns, & Read [10]. Oxygen stable isotope ratios were analyzed with thermal conversion through pyrolysis (HTO, HEKAtech, Wegberg, Germany) coupled to a continuous flow isotope-ratio mass spectrometer (delta V advantage, Thermo Fisher Scientific) via a ConFlo IV open-split interface (Thermo Fisher Scientific) as described by Gebauer, Preiss, & Gebauer [11]. Isotope abundances are given in δ values, calculated according to the following equation:

(2) $$\delta^{13}C,\delta^{15}N,\;\mathrm{or}\;\delta^{18}O=\left(R_{\mathrm{sample}}/R_{\mathrm{standard}}-1\right)\times 1000\;\left(‰\right)$$

where R_sample_ and R_standard_ are the ratios of heavy to light isotope of the samples and the respective standard. This so called “delta notation” is used to express the difference between the isotope-number ratio of an unknown sample and an internationally agreed zero-point with known isotopic composition. Nowadays, it is commonly used in many fields of isotope research, as it enables a more convenient and accurate expression of small differences in isotope-number ratios that are usually in the order of 10^−3^ or smaller. The standard gases (Riessner-Gase, Lichtenfels, Germany) were calibrated with respect to international standards (CO_2_ vs. V-PDB, N_2_ vs. N_2_ in air, CO vs. V-SMOW) with the reference substances IAEA-CH-6, IAEA-CO-8 and NBS 18 for the C isotopes, N1 and N2 for the N isotopes and IAEA601 and IAEA602 for the O isotopes, all provided by the IAEA (International Atomic Energy Agency, Vienna, Austria). Measurements of the laboratory standard acetanilide were routinely performed to control the reproducibility of C and N isotope abundance analysis [12]. Acetanilide was monitored with variable sample weight at least six times per batch of 50 samples. For oxygen isotope abundance measurements, benzoic acid was analyzed with variable sample weight at least six times within each run of 40 samples. The uncertainty of measurements was always below 0.2‰ for δ^13^C, δ^15^N and 0.6‰ for δ^18^O. Data evaluation was performed in the software ISODAT (Version 2.0, Thermo Fisher Scientific).

### 2.4. Fourier-Transform Near-Infrared Spectroscopy (FT-NIR)

FT-NIR measurements were performed on a Bruker MPA spectrometer (Bruker Optik GmbH, Ettlingen, Germany) equipped with an interferometer, an integrating sphere for diffused reflection and a lead sulfide (PbS) detector. The sample cup was filled to the brim with whole grains and placed on a sample rotator. Spectra were recorded in triplicates using 64 scans in the range of 12,500 to 3600 cm^−1^ at a resolution of 16 cm^−1^. Data were collected and preprocessed applying baseline correction using the concave rubber-band method with 10 iterations on 64 baseline points in OPUS (Bruker Optik GmbH). Analysis of FT-NIR spectra was limited to the range between 11,000 to 4000 cm^−1^, and therefore excluded the flanking regions that display a strongly reduced signal-to-noise ratio.

### 2.5. Proton Nuclear Magnetic Resonance (^1^H-NMR)

For ^1^H-NMR measurements, grains were ground to wholemeal flour in a ball mill (MM400, Retsch GmbH) at 30 Hz for 2 min. 50 mg of wholemeal flour were weighed in a 2 mL reaction tube (Carl Roth GmbH, Karlsruhe, Germany) and 2 mL of HPLC-grade water was added. The sample was extracted for 2 h in an overhead shaker (Tube Revolver D-6050, neoLab Migge GmbH, Heidelberg, Germany) at 40 rpm before centrifugation for 45 min at 13,300 rpm (MIKRO 185, Andreas Hettich GmbH, Tuttlingen, Germany). 900 µL of the supernatant was transferred in a 2 mL reaction tube (Carl Roth GmbH) and 100 µL of buffer was added [13]. The pH was adjusted to 3.10 (± 0.02) using an automated titration system (BTpH Unit, Bruker BioSpin, Rheinstetten, Germany). The sample was centrifuged again for 45 min at 13,300 rpm (Andreas Hettich GmbH) and 600 µL of the supernatant was transferred in a 5 mm NMR tube. A 400 MHz FoodScreener^TM^ spectrometer equipped with a BBI probe head with z gradients was used to acquire the spectra (Bruker Biospin) with a spectral width of 20.458 ppm. All samples were handled with the IconNMR and TopSpin 3.5 software. A 1D-NOESY-experiment (noesyggpr1d) was performed with an acquisition time of 4 s and 32 scans. The excitation pulse was determined before each measurement and the temperature was set to 300 K. The receiver gain was set to a constant value of 32. An exponential window function with an LB of 0.3 was applied during automatic processing. Spectra were referenced to the signal of the internal standard 3-(trimethylsilyl)-propanoic acid-*d*_4_ sodium salt (TSP, δ = 0 ppm). To ensure the qualitative comparability of the barley flour extracts, the recovery rate of at least 95% was determined using the signal of the compound betaine, which was found to be a promising marker for drought response of barley in the course of this study, after double extraction and measurement of a representative sample.

### 2.6. Data Preprocessing and Statistical Analysis

Plots for temperature and precipitation data were done in R version 3.5.3 using additional packages (ggplot2, ggridges, RColorBrewer). Maps displaying the aridity index grids with a spatial resolution of 1 km × 1 km were reproduced graphically in the open-source software QGIS version 3.1 (Quantum Geographic Information System). Univariate statistical analysis of isotope data was also conducted in the computing environment R. As the assumptions for the use of a parametric test were not met, differences between groups were evaluated by a non-parametric two-sided Mann–Whitney–Wilcoxon test (α = 0.05). Chemometric analysis (Kruskal–Wallis test, PCA) of FT-NIR spectra and combined data were performed in MATLAB (Version R2019a, TheMathWorks, Natick, MA, USA). Dimensionality reduction was achieved by splitting the FT-NIR-spectra into bins. Binning (also called bucketing) is a very common pre-processing step for spectral data, that often contain thousands of data points/variables. To ease statistical analysis, the number of variables is reduced by dividing each spectrum into for example equidistant (uniform) so-called bins/buckets and calculating the integral (total area within each bucket) of each bin. In our particular case, each FT-NIR spectrum was divided into 300 equidistant regions in the range between 11,000 and 4000 cm^−1^. Afterwards, the IRMS variables (δ^13^C, δ^15^N, δ^18^O) were attached to the data set. Feature selection was performed for combined data, extracting the 50 most relevant variables based on ranked class-related variance using a Kruskal–Wallis test. Specifically, the test statistic (*H* statistic) of the Kruskal–Wallis test was calculated and used as a filter score, where higher values of the test statistic indicate that the values of the corresponding variable differ more between the classes. Subsequently, the test statistic values were sorted from the highest value to the lowest value to reveal the most relevant variables. Even if statistical approaches are sophisticated, they should not be blindly relied upon. For this reason, we looked at the extracted variables manually to verify that they indeed belong to meaningful spectral regions rather than, e.g., noise. Here, all of the 50 extracted variables were found to be relevant, hence they were used for further statistical analysis. Selected features were then autoscaled (mean-centering followed by division of each column/variable by the standard deviation of that column) and processed using principal component analysis (PCA), to obtain an impression of the differences between spectra among harvest years. As PCA is an exploratory technique rather than a classification technique, we defined a criterium based on which a sample is assigned to a class. Here, we have classified the observations according to their position in the PCA score subspace by defining a threshold based on Euclidean distance to the calculated center of each group at a 95% confidence level (α = 5%). Using this classification technique, model validation using Monte Carlo cross-validation was performed. Specifically, 100 random models were generated with 90% of the data set as training data and the remaining 10% as test set.

## 3. Results and Discussion

### 3.1. Weather at the Sampling Site Differed between 2017 and 2018

**Figure 1:**
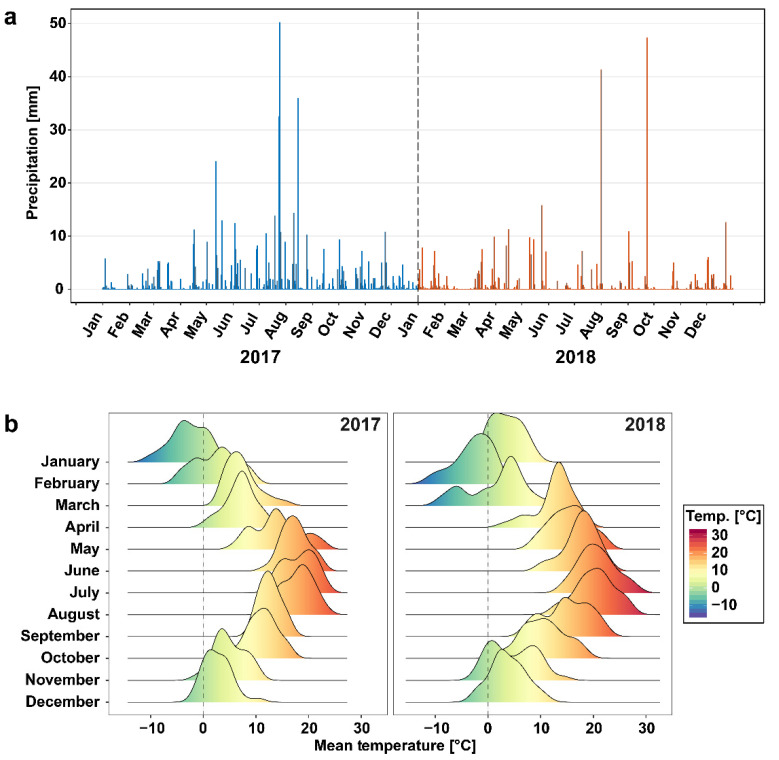
Precipitation and temperature at the sampling site. (**a**) The graph displays the daily precipitation totals (in mm) at the sampling site for the crop years 2017 (blue) and 2018 (red). (**b**) Density plots show the distribution of daily average air temperatures (in °C) for the crop years 2017 (left) and 2018 (right). Raw data were retrieved from the weather station (Erfurt-Weimar, German Meteorological Service) with the closest proximity to the sample origin.

Total precipitation at the sampling site in 2017 was 573.9 L/m^2^, which corresponds to 106% of the mean of the 30-year reference period (1981–2010). In comparison, total precipitation in 2018 was 70% of the mean of the reference period with a total of 377.1 L/m^2^. During the three summer months (June–August), the sum of rainfall was 285.1 L/m^2^ (155%) in 2017, while in 2018, only 77 L/m^2^ were recorded, meeting 42% of the reference period mean (Figure 1a). In addition, the 2018 summer months had 48 days with maximum temperatures above 25 °C (2017: 28 days), which very likely led to co-occurring heat and drought stress (Figure 1b).

**Figure 2:**
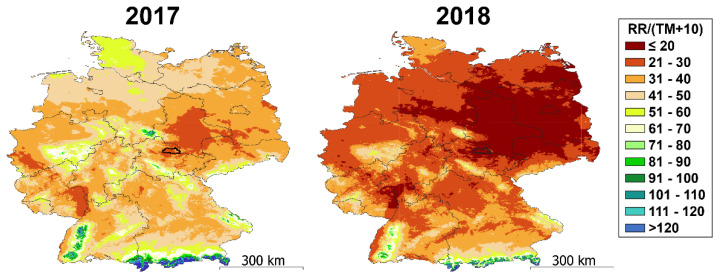
Comparison of the aridity in Germany. Maps show the spatial variation in annual drought index (De Martonne aridity index, I_DM_) for Germany in the years 2017 (left) and 2018 (right) and the map scale. All values are calculated using the formula: RR/(TM + 10), where RR is the annual sum of precipitation (in mm) and TM is the annual mean air temperature (in °C). Lower index values refer to more pronounced aridity at respective location. The sample origin is highlighted with a thick line and shows index values below 30 in 2017 (humid to Mediterranean) and values below 20 in 2018 (semi-dry to dry).

Recent findings showed that most barley varieties are particularly sensitive to a combination of these stresses in comparison to heat or drought individually [14,15]. Both precipitation and temperature are included in the calculation of the De Martonne’s aridity index (I_DM_), which was plotted on a map of Germany. Based on the classes of this frequently used index, the barley experienced humid to Mediterranean (I_DM_ ≤ 30) conditions in 2017. In contrast, the year 2018 in the region the samples were sourced was characterized by semi-dry to dry conditions (I_DM_ ≤ 20) [9] (Figure 2).

### 3.2. Stable Isotope Abundances Indicate Severe Drought Stress

**Figure 3:**
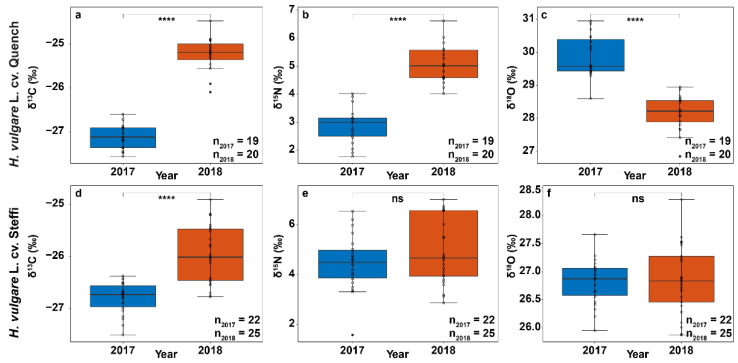
Natural stable isotope abundances of the analyzed spring barley samples. Box and whisker plot graphs with interquartile ranges, medians, error bars (non-outlier minimum and maximum) and results of the Mann–Whitney–Wilcoxon test on the isotopic values (δ^13^C, δ^15^N, δ^18^O) of spring barley samples from the two varieties (**a**–**c**) Quench and (**d**–**f**) Steffi. Samples are grouped based on the crop years 2017 (blue) and 2018 (red). Highly significant differences (*p* < 0.0001) are highlighted with four stars (****), while non-significant differences are marked with ‘ns’. Outliers are denoted as filled circles.

Carbon isotope abundances of all analyzed barley samples ranged from −24.5‰ to −27.6‰ with a mean of −26.3‰, which perfectly fits the typical average ratio for the C_3_ crop barley of −25.9 ± 0.7‰ [16]. Samples from the harvest year 2018 showed significantly less negative δ^13^C values for both Quench (Mdn, N_2017_ = −27.1‰, *n* = 19; Mdn, N_2018_ = −25.2‰, *n* = 20; U = 0, *p* < 0.0001) and Steffi (Mdn, N_2017_ = −26.7‰, *n* = 22; Mdn, N_2018_ = −26.0‰, *n* = 25; U = 52.5, *p* < 0.0001) compared to samples from 2017 with a more pronounced difference for the Quench variety. The results match the values of studies from Ferrio et al., who found less negative δ^13^C values for bread wheat kernels grown under increased drought stress [17]. Between the two trials that varied the most in water availability, they observed a difference in carbon isotope composition of 2.2‰. The less negative values in kernels grown under heavy drought stress match our expectations as the relationship between water status and the carbon isotopic composition of plants and the underlying mechanism are well researched [18]. During assimilation of CO_2_, plants utilizing the C_3_ photosynthetic pathway (e.g., barley) discriminate against the heavy isotope of carbon (^13^C). Under normal conditions, the CO_2_-fixing enzyme ribulose 1,5-bisphosphate carboxylase/oxygenase (RubisCO) favors the light ^12^CO_2_. Stomatal closure in response to drought stress reduces water loss caused by transpiration but also leads to CO_2_ being ‘trapped’ inside the leaf’s internal air spaces. The closed stomata force the enzyme to process more of the heavy ^13^CO_2_, as the trapped CO_2_ is recycled [19,20]. The resulting change in isotopic composition of the biomass can be detected in various parts of crops but appears to be most pronounced in mature grains, as they might serve as a sink for different photosynthetic plant organs [21]. Compared to Quench, which is a high-yielding barley cultivar, Steffi is a rather old and less cultivated variety for malting. Therefore, differences in genotype and reaction to drought might have attributed to the more pronounced difference in ^13^C abundance for Quench (Figure 3a,d) [22].

In fact, there is growing evidence that some barley genotypes, which carry more desirable wild-type traits, have a higher abiotic stress tolerance to environmental factors such as drought and heat, thus raising interest in targeted breeding of cultivated barley, especially in view of the challenges posed by climate change [23,24]. The reported N isotopic compositions showed significant interannual differences for the Quench variety (Mdn, N_2017_ = 3.0‰, *n* = 19; Mdn, N_2018_ = 5.0‰, *n* = 20; U = 1, *p* < 0.0001) with a higher δ^15^N in the dry year 2018, while no difference was found for Steffi (Mdn, N_2017_ = 4.5‰, *n* = 22; Mdn, N_2018_ = 4.7‰, *n* = 25; U = 229.5, *p* > 0.05) (Figure 3). The connection between plant δ^15^N values and aridity are not straightforward, as multiple factors such as microorganism activity, N source, and plant metabolism influence the signature. The observed increase in δ^15^N for Quench might be attributed to differences in agricultural practice, response of nitrate reductase activity to drought or due to limited uptake and transfer of N to the upper plant parts [25]. Apart from the information that the barley comes from conventional cultivation, details on fertilization are missing, which makes interpretation of the δ^15^N values rather challenging. At the same time, such obstacles reflect the intention of the study, namely to create real conditions that can be transferred to the market. In our approach, these uncertainties can, to some extent, be compensated by using several measurement techniques for a more holistic point of view. As for nitrogen isotope composition, δ^18^O was significantly different between the two harvest years for the variety Quench (Mdn, N_2017_ = 29.6‰, *n* = 19; Mdn, N_2018_ = 28.2‰, *n* = 20; U = 376, *p* < 0.0001) with lower δ^18^O values in 2018, while no significant difference was found for the Steffi variety (Mdn, N_2017_ = 26.9‰, *n* = 22; Mdn, N_2018_ = 26.8‰, *n* = 25; U = 270, *p* > 0.05) (Figure 3). A missing interannual difference in δ^18^O values is rather unexpected as similar to δ^13^C values, oxygen isotopic signatures are expected to increase with higher evaporative demand and lower stomatal conductance [17]. However, several studies suggest that there is a lack of a clear relationship in grains compared to, e.g., leaf material or whole plant material. For spring wheat, Barbour, Fischer, Sayre, & Farquhar reported a significant positive correlation of δ^13^C and δ^18^O in flag leaves, but the relationship was less clear in grains [26]. We conclude, while grain δ^13^C seems to be a good marker for water status during grain filling, other factors seem to specifically affect δ^18^O. The signal could be dominated by variations in δ^18^O signature of the source water, or it could be influenced by biochemical fractionation during synthesis of grain organic matter from remobilized assimilates [17]. Considering the heavy drought in 2018, a higher contribution of pre-anthesis reserves to grain filling is very likely, which would lead to lower δ^18^O values [27,28].

### 3.3. FT-NIR Spectra Show Additional Drought-Related Differences

**Figure 4:**
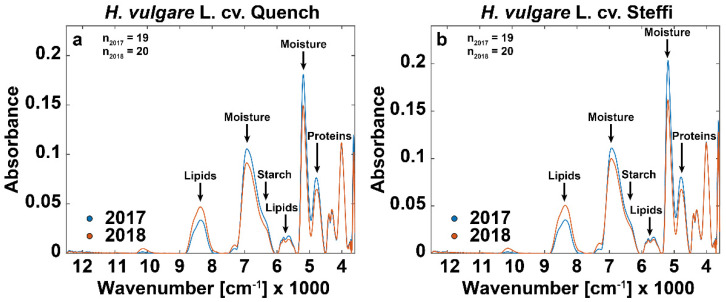
Comparison of spring barley kernel FT-NIR spectra. Figures display baseline-corrected average FT-NIR spectra (4000 to 12,000 cm^−1^) for whole kernels of the spring barley varieties (**a**) Quench and (**b**) Steffi. Samples are grouped based on the crop years 2017 (blue) and 2018 (red). Assignments of relevant spectral bands are indicated at the respective absorbance peak.

For visual presentation of the differences in the FT-NIR spectra between the samples from the two harvest years, average spectra were calculated, and signal assignments of fundamental regions were included (Figure 4).

It is noteworthy that the spectra of both cultivars show the same changes for the year 2018, indicating similar metabolite responses to drought stress. The absorbance peak at around 8264 cm^−1^ (1200 nm) was related to the second overtone of C-H stretching, whereas the absorbance peak at 5665 cm^−1^ (1765 nm) was related to the first harmonic of C-H stretching. Both peaks are attributed with lipids and showed noteworthy differences between the harvest years, which is consistent with results from a study using GC/MS-based metabolite profiling [29,30,31]. Further, we observed two absorbance bands with outstanding separation between harvest years that were linked to moisture at around 6800 cm^−1^ (1470 nm, first overtone of O-H stretching) [29,32,33] and 5170 cm^−1^ (1934 nm, O-H stretching) [29,30,32,34,35]. Based on a nationwide report by the Federal Ministry of Food and Agriculture, in 2018 all cereals, including barley, showed significant reduction in average moisture content in comparison to the previous year, which fits our observations [36]. The absorbance band around 6300 cm^−1^ (1587 nm, first overtone of O-H stretching) is a bit lower for the grain samples from 2018 and was associated with starch, whose synthesis is known to be hindered by drought stress [29,30,34,37]. Finally, we could observe differences between harvest years at the absorbance peak around 4854 cm^−1^ (2060 nm). The band is associated with a combination of C-H stretching, N-H stretching, and O-H stretching and is related to variation in protein characteristics potentially attributed to osmotic adjustments [29,31,32,34].

### 3.4. Data Fusion Enables Differentiation of Barley Harvest Years

**Figure 5:**
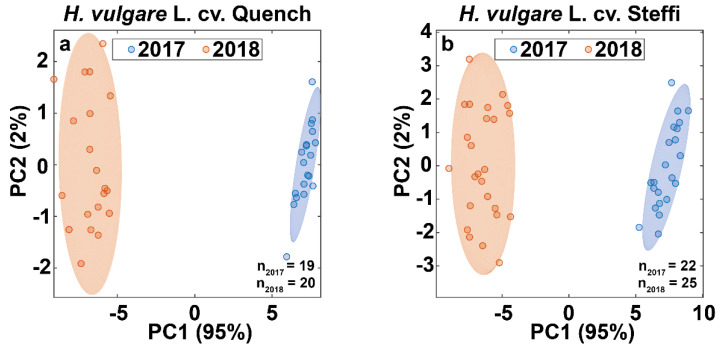
Chemometric analysis of combined FT-NIR and IRMS data. Score plots including 95% confidence ellipses of the first two principal components from the PCA of fused data (FT-NIR and IRMS). Plots are displayed for the two spring barley varieties (**a**) Quench and (**b**) Steffi, and the samples are colored by the crop years 2017 (blue) and 2018 (red). For each principal component, the respective percentage of variance explained is given in the axis label.

In a next step, we statistically evaluated the FT-NIR spectra and data produced by IRMS, using Kruskal–Wallis tests and principal component analysis (PCA). The created models achieved 100% correct classification of harvest years for both barley varieties (Figure 5).

To gain insight into which of the included variables contributed most to the separation, we ranked them by importance using a Kruskal–Wallis test. We found that the abundance peak at around 8264 cm^−1^ (1200 nm) was the most important, followed by the absorbance bands around 4854 cm^−1^ (2060 nm), 5170 cm^−1^ (1934 nm), 5665 cm^−1^ (1765 nm), and 6800 cm^−1^ (1470 nm). This is consistent with findings from the literature, which state that the composition and content of lipids and proteins in barley change significantly under stress conditions such as heat and drought [24,38]. Interestingly, in the statistical analysis of the Quench variety, the carbon isotopic composition appeared among the most important variables for the separation of crop years, but not for the Steffi variety. Given that the difference in the natural ^13^C abundance is a marker for water-use efficiency, there is growing evidence that the old Steffi variety is better adapted to drought [20].

### 3.5. ^1^H-NMR Confirms Observations by Means of the Marker Betaine

**Figure 6:**
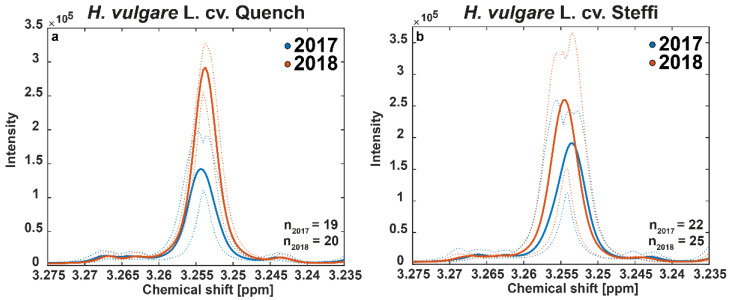
Comparison of the content of the plant-physiological marker betaine. Plots show the ^1^H-NMR spectra region (3.275–3.235 ppm), including the signal of betaine at 3.255 ppm chemical shift. The two spring barley varieties (**a**) Quench and (**b**) Steffi were analyzed as wholemeal flour extracts. Average spectra are displayed as solid lines and the 95% confidence intervals are represented as dotted lines. Samples are colored by the harvest years 2017 (blue) and 2018 (red). Note that there are minor differences in chemical shift due to slight variations in the sample conditions, which lead to apparent multiplets when the included spectra are averaged. This also creates multiple maxima for the confidence intervals (dotted lines). However, the betaine signal at 3.255 ppm is a singlet.

Finally, we used ^1^H-NMR spectra of sample extracts that serve as quantitative metabolite fingerprints. These can not only be analyzed in a non-targeted approach, but also in high-resolution identification of molecular markers. In our case, we focused on plant physiological markers such as osmolytes, which might indicate a plant’s response to stress conditions. A direct comparison of the ^1^H-NMR spectra from the two investigated harvest years revealed a significantly increased signal intensity at 3.255 ppm chemical shift in all samples from the year 2018. Importantly, this is regardless of which of the two barley varieties was considered (Figure 6).

A comparison with common databases and subsequent spiking experiments for confirmation with the pure substance revealed that the signal refers to betaine (trimethylglycine). Accumulation of this methyl donor is a well-known and critical response of barley to environmental stress such as drought [39]. We would like to mention here that for the purposes of this paper, we consider only this section of the spectrum, as it provides the marker for drought stress and thus the biological background. However, it can be assumed that an elaborate statistical analysis of the complete NMR spectra could reveal other exciting relationships. On a molecular level, the connection between drought stress and betaine accumulation can be explained by the function of the plant hormone abscisic acid (ABA), which not only induces stomatal closure to prevent water loss but was also found to increase expression of betaine aldehyde dehydrogenase (BADH), catalyzing the last step in the synthesis of betaine [40]. The relationship is underpinned by the fractionation we observed for the carbon isotopic composition, which is caused by stomata regulation. Although this important plant physiological process has often been demonstrated by experiments with whole plants or leaves, elevated concentrations of betaine resulting from high temperatures and drought have also been observed in studies with grains of wheat and barley [31,41]. In respective results, there were clear differences between individual genotypes, on the one hand in the basic content of betaine, but also in the degree of accumulation in response to stress. This might explain the difference observed between the two investigated varieties, with the old cultivar once more showing a milder response, possibly due to a higher tolerance to environmental stress.

## 4. Conclusions

Global warming creates circumstances for the agricultural sector that require rapid action and unprecedented scientific approaches. A central component is the transfer of knowledge from controlled field experiments to real market samples. Our results show that spring barley undergoes noticeable changes in its chemical composition in an extremely dry and hot year like 2018, enabling a solid discriminability from samples of 2017. The significantly less negative δ^13^C values for both analyzed varieties proved to be a suitable marker for water status during grain filling. Furthermore, FT-NIR and ^1^H-NMR spectra showed significant differences between the two harvest years, especially in the absorbance bands related to lipids and proteins and the stress marker betaine. All observed chemical changes could be explained and linked by means of plant physiology, which is an integral part of authenticity testing of plant-based foods. Furthermore, the weaker stress response of the old cultivar “Steffi” has shown that the data obtained can also be transferred to targeted breeding of varieties that tolerate future climate conditions. We are convinced that additional research on our initial findings will show the broad applicability to unknown systems and will be the go-to practice in agricultural science.

## Data Availability

All weather-related data can be accessed from the Climate Data Center, provided by the German Meteorological Service (https://cdc.dwd.de/portal), https://opendata.dwd.de/climate_environment/CDC/). The MATLAB code used for the chemometric analysis of data is available at the gitlab repository of the co-author Felix Rüll (https://gitlab.com/FelR/ai-omics). Detailed raw data for IRMS, FT-NIR and ^1^H-NMR measurements are available upon request from the corresponding author.
